# Preventive and Therapeutic Strategies for Bovine Leukemia Virus: Lessons for HTLV

**DOI:** 10.3390/v3071210

**Published:** 2011-07-19

**Authors:** Sabrina M. Rodríguez, Arnaud Florins, Nicolas Gillet, Alix de Brogniez, María Teresa Sánchez-Alcaraz, Mathieu Boxus, Fanny Boulanger, Gerónimo Gutiérrez, Karina Trono, Irene Alvarez, Lucas Vagnoni, Luc Willems

**Affiliations:** 1 Molecular and Cellular Epigenetics, Interdisciplinary Cluster for Applied Genoproteomics (GIGA), University of Liège (ULg), 4000, Liège, Belgium; E-Mails: Sabrina.Rodriguez@ulg.ac.be (S.M.R.); n.gillet@ulg.ac.be (N.G.); fanny.boulanger@hotmail.fr (F.B.); 2 Molecular and Cellular Biology, Gembloux Agro-Bio Tech, University of Liège (ULg), 5030, Gembloux, Belgium; E-Mails: afflorins@ulg.ac.be (A.F.); Alix.deBrogniez@student.ulg.ac.be (A.d.B.); mteresasanchez@hotmail.com (M.T.S.-A.); Mathieu.Boxus@ulg.ac.be (M.B.); 3 Instituto de Virología, Centro de Investigaciones en Ciencias Veterinarias y Agronómicas, INTA, C.C. 1712, Castelar, Argentina; E-Mails: ggutierrez@cnia.inta.gov.ar (G.G.); ktrono@cnia.inta.gov.ar (K.T.); ialvarez@cnia.inta.gov.ar (I.A.); lvagnoni@cnia.inta.gov.ar (L.V.)

**Keywords:** BLV, therapeutics, vaccines, epigenetic, eradication, control

## Abstract

Bovine leukemia virus (BLV) is a retrovirus closely related to the human T-lymphotropic virus type 1 (HTLV-1). BLV is a major animal health problem worldwide causing important economic losses. A series of attempts were developed to reduce prevalence, chiefly by eradication of infected cattle, segregation of BLV-free animals and vaccination. Although having been instrumental in regions such as the EU, these strategies were unsuccessful elsewhere mainly due to economic costs, management restrictions and lack of an efficient vaccine. This review, which summarizes the different attempts previously developed to decrease seroprevalence of BLV, may be informative for management of HTLV-1 infection. We also propose a new approach based on competitive infection with virus deletants aiming at reducing proviral loads.

## 1. Introduction

As early as in the 19th century, a series of reports already described the occurrence of clinical signs associated with enzootic bovine leukosis (EBL). Initial observations from Leisering of yellowish nodules in the enlarged spleen of a leukocytic cow were communicated in the German literature in 1871 ([1], cited by [2]). Three years later, Bollinger described bovine leukemia as a well-defined clinical entity, and in 1876 Siedamgrotzky and Hofmeister recorded the first cases of bovine lymphocytic malignancies ([3,4]; cited by [2]). Thus, Europe, and more specifically the Memel area, then regarded as East Prussia and now located in the contemporary Lithuania, might be thought of as the cradle of enzootic bovine leukemia ([5]; cited by [6]). Diffusion and wide spread of the disease occurred by the introduction of European cattle breeds in countries free of the disease. Despite all these pieces of evidence, the infectious nature of EBL etiological agent was discovered many decades later based on epidemiological evidence [7]. Finally, the causative agent of this malignant disease was isolated in culture in 1969 [8] and designated bovine leukemia virus (BLV).

BLV is an oncogenic B-lymphocytotropic retrovirus that infects cattle inducing a persistent infection with diverse outcomes [9–16]. The great majority of BLV-infected animals (around 70%) are asymptomatic carriers of the virus. In these animals, neither clinical symptoms nor alteration of the total lymphocyte count are evidenced. Thus, they can only be identified by the presence of anti-BLV antibodies and/or of proviral DNA [14,16]. Approximately, one-third of BLV-infected bovines develop a benign polyclonal proliferation of B cells called persistent lymphocytosis (PL). This clinical condition is characterized by an increase in the absolute number of peripheral blood circulating B lymphocytes associated with an inversion of the B/T lymphocyte ratio [9,14,17,18]. Despite these hematologic alterations, PL animals do not develop any other apparent clinical signs. PL is usually stable for several years but can also progress to the tumor phase [9,14,18].

The most conspicuous clinical manifestation of BLV infection is the development of lymphoid tumors. Fatal lymphoma or lymphosarcoma (LS) occurs in less than 5–10% of infected animals, predominantly adult cattle older than 4–5 years old [10,14]. The development of tumors is not necessarily preceded by a phase of PL, but this is the case in two-thirds of the animals [14,19]. Unlike persistent lymphocytosis, B-cell expansion is of mono- or oligo-clonal origin [13,14,20–24]. Local proliferation of B cells, called lymphosarcoma, can occur within different organs and tissues leading to a series of defects that are finally incompatible with the survival of the animal. In addition, transformed B cells can also induce the enlargement of lymph nodes and cause lymphoma [10,14]. Besides an impact on survival, BLV infection also impairs the immune system leading to opportunistic infections [25–27].

## 2. Transmission

BLV is transmitted horizontally, essentially through the transfer of infected cells [28]. Since free virus is very unstable, BLV-infected cells (B-lymphocytes, monocytes/macrophages, *etc.*) present in blood or milk appear to be the best vehicles of natural transmission [16]. Due to the high number of infected cells they contain, animals with PL are particularly efficient in transmitting the virus [28]. In herds, iatrogenic procedures largely account for the propagation of the infection. Cattle management procedures which involve transfer of infected blood (*i.e.*, dehorning, ear tattooing, rectal palpation, and, essentially, the use of infected needles) were postulated as a common mode of transmission [29]. In addition, prolonged direct contact between infected and healthy animals has also been considered as a risk factor for BLV transmission. Transfer of infected blood might also occur in regions with high density of hematophagous insects [30,31]. Perinatal or postnatal transmission of BLV frequently happens in herd conditions. The rate of transmission *in utero* varies between 4 and 18% [32–34], with highest risk in calves born from cows with PL [35]. Although BLV transmission has been postulated to occur from cow to calves via milk, this route of infection was only demonstrated experimentally [36–38]. Despite the presence of BLV proviral DNA in colostrum and milk from infected cows, calves can remain uninfected over extended periods of time likely due to the protective role of maternal antibodies present in the colostrum [39–41].

## 3. Epidemiology

Sero-epidemiological surveys have shown that BLV infection is widespread in all continents except in Europe. Efforts in the implementation of control measures and campaigns to eradicate BLV infection in Western Europe have been successful [42–45]. Recently, the European Economic Community (EEC) declared most of its member states as officially free of EBL. In contrast, the situation is different in Eastern Europe where the disease is still present in several countries (Bulgaria, Croatia, Estonia, Latvia, Poland, Romania, Ukraine) [46].

Similar attempts to eradicate BLV infection in Australia and New Zealand dairy herds began in the mid-1990s. More than 98% of dairy herds were negative in 2005 [47,48].

Detailed information about the epidemiological situation in the United States of America was collected through the National Animal Health Monitoring (NAHMS). Survey studies in 2007 revealed that 83.9% of U.S. dairy herds were positive for BLV [49]. Recent epidemiological data is available for several provinces in Canada. All agree in indicating high seroprevalence rates reaching up to 89% and 20.8%–37.4% at herd and individual levels, respectively [50–54].

In South America, individual infection rates between 34 and 50% were reported in Colombia, Venezuela, Chile and Uruguay [55–58]. In Argentina, individual and herd prevalence levels scale up to 32.8% and 84%, respectively [59]. In Brazil, the individual prevalence of BLV infection varies considerably among states and reaches levels beyond 50% [60–67].

The epidemiological situation in Asia is more uncertain. The International Organization of Epizootics (OIE) recognizes that BLV is present in Indonesia, Taipei (China) and Mongolia [46]. Surprisingly, only about 5% of animals were positive for BLV in Cambodia and Taiwan [68,69]. The seroprevalence rates in Japan were found to be 28.6% and 68.1% at the individual and herd levels, respectively [70]. In Korea, individual seroprevalence rates exceeded 50% whilst 86.8% of dairy herds were infected [71].

Regarding the Middle East countries, reports indicate that the prevalence of BLV infection is somewhat lower than in other regions of the world, *i.e.*, about 20% [27,72–74]. Exceptions in this region include Turkey and Iran where the herd seroprevalence levels climb to 48.3% and 64.7%, respectively, while the individual seroprevalence in Iran was estimated between 17 and 24.6% [75–77], although some argue that prevalence levels are lower [78].

## 4. Preventive and Therapeutic Strategies

Understanding the mechanisms of pathogenesis has been instrumental for developing therapeutic and preventive strategies against BLV infection. The different approaches that were developed are reviewed in this section.

### 4.1. Removal or Segregation Approaches

#### 4.1.1. Test and Eliminate

A first strategy is to identify and eliminate BLV-infected cattle. This approach requires identification of BLV-positive animals either by hematological, genomic or serological methods, immediate removal of positive cases from the herd and, finally, prompt slaughtering [7,79,80].

This “test and elimination” methodology has been instrumental in accomplishing the BLV-free status at herd and regional scales within a relatively brief period of time when compared to other alternatives [7,43–45,81–89]. Evidence of the efficiency of this strategy is illustrated by the successful eradication of the disease in several countries of Western Europe.

Although this approach has been efficient, its feasibility faces some important restrictions. A key limitation is that the initial prevalence rate of infection should not be high due to its very high economic cost. Hence, this strategy can be particularly justified in pedigree breeds having high genetic potential as well as for export to BLV-free countries. Nevertheless, even when the rates of infection are low, the “test and eliminate” approach is a burdensome strategy due to diagnostics, premature culling and replacement of reactors. Loss of genetic and reproductive potential must also be considered.

In fact, this strategy requires governmental economic compensation policies to be successful. If no official subsidizing action is adopted by the local authorities, the costs of implementation of such a strategy quickly exceeds the potential benefits. Countries such as USA, Canada, Argentina, and Japan lacking financial compensatory policies usually failed to obtain adherence to enroll in these programs [49–54,59,70,90,91].

To summarize, the pros and cons of the “test and eliminate” strategy are outlined in Table 1.

#### 4.1.2. Test and Segregate

Another approach aims at reducing part of these costs by segregating instead of culling infected animals. Control programs based on segregation require detection of seropositive animals and confinement of BLV-infected and seronegative herds in strictly separated areas [28,92,93]. It has been proposed that a minimum distance of 200 meters must separate the two herds to avoid transmission [92]. An alternative option is to keep the animals in the same farm and to manage them separately. For optimal results with any of these options, separate equipment or at least careful disinfection and hygiene of non-disposable equipment should be implemented (Table 1). The main advantage of this approach is the reduction of costly losses due to compulsory premature culling and replacement of BLV-positive animals. Although quite demanding, this type of program has been useful to decrease prevalence or even achieve eradication of the disease [84,92,94]. The disadvantage is that there is a permanent risk of reintroducing infected animals and, therefore, it may be slower than the ‘test and eliminate’ option [95].

The removal/segregation approaches both require specific and regular identification of infected animals. Discrimination of BLV-infected animals from the herd was originally performed by hematologic screening based on leukocyte counts [7]. Later on, more sensitive and specific techniques (*i.e.*, AGID, agar gel-immunodiffusion test and ELISA, enzyme-linked immunosorbent assay) became routine diagnostic methods. These serologic assays performed on serum or milk were widely used and became the official tests recommended for international trade [96]. However, these tests can be misleading since the presence of anti-BLV antibodies does not necessarily imply that the animals are infected and *vice versa*. Indeed, there is a “latency” period of several weeks between infection and seroconversion [97]. Also, calves can have anti-BLV antibodies due to maternal antibodies from the colostrum [33,36,39,98,99] or to parturition [99]. A significant fraction of these calves will remain uninfected although they carry anti-BLV antibodies.

Therefore, a more accurate way to monitor BLV infection is PCR [100,101]. BLV-infected cattle can further be classified according to their proviral load (PVL) [102]. In fact, the proviral load in the peripheral blood is a predictive marker of transmission risk. Thus, PVL can be used as a criterion to discriminate and eliminate/segregate highly infected cows [102–104]. Besides technical challenges (contaminations), the disadvantages of PCR-based diagnostics are higher costs and the need of more complex equipment.

### 4.2. Corrective Management and Veterinary Practices

This type of approach aims at limiting transfer of BLV-infected cells present in blood, milk, secretions, excretions, syringes or surgical instruments.

Among the diverse control measures that can be implemented in a preventive or corrective management control plan, the following constitute essential practices:

- (i) use of individual, single-use needles and syringes during vaccination or therapeutic protocols;
- (ii) use of individual, single-use obstetrical sleeves (or at least replacement between examination of BLV-reactors and non-infected animals);
- (iii) use of disposable equipment (or at least cleaning, disinfection or sterilization of reusable materials and surgical instruments) in procedures such as dehorning, tattooing, implanting, cauterizing, castration or ear-tagging;
- (iv) use of electrical or gas burning devices rather than gouging equipment during dehorning;
- (v) feeding calves with colostrum or whole milk from non-infected dams, pasteurized colostrum from BLV-infected cows or milk replacer;
- (vi) elimination of insects, particularly in densely populated farm areas (milking areas, free-stalls, barns) in order to minimize potential transmission between animals through arthropod vectors;
- (vii) natural and/or artificial insemination and embryo transfer with BLV-free dams and bulls.

Other potentially beneficial management control measures might include: (i) prevent introduction of infected animals into the herd by testing and quarantining/isolating newcomers; (ii) separate animals by age to decrease contacts (iii) minimize movement of animals between milking/feeding groups; (iv) use individual calf hutches for newborn calves; and (v) limit access to outside visitors.

Compared to the test and eliminate/segregate strategies, the “corrective management and veterinary practices” approach is clearly more cost-effective. Neither investments on facilities nor removal of infected cattle nor constant surveillance of the herd serological status are required. The disadvantages of the corrective management approach are that practices are intensively laborious and vulnerable to environmental and human variables. In this regard, careful appraisal of the effort required before the enrolment into a corrective management control plan must be considered. Strict compliance to the rigorous measures should be enforced and a long-term engagement with the program is required until fruitful benefits of this strategy are observed (see Table 1). In addition, adequate training of personnel and farm employees on BLV prevention and biosecure practices might be considered. The efficacy of a “corrective management and veterinary practices” strategy based exclusively on strict sanitary measures is still controversial [104–106].

### 4.3. Selection of BLV-Resistant Cattle

Livestock breeding programs exploit selection of genetic traits beneficial for production (e.g., milk yield, growth, reproduction) [107,108]. In principle, it would be possible to select breeds that are less susceptible or even resistant to BLV infection. Immune responsiveness and heritable resistance or susceptibility to infection are influenced by the host major histocompatibilty complex (MHC) [107–110]. The Bovine Lymphocyte Antigen (BoLA) refers to the major histocompatibilty complex in bovine species (*Bos taurus* and *Bos indicus*) [111–113]. Pioneering work suggested that host genetics factors determined cattle susceptibility to BLV infection [114]. Early studies initially attributed a linkage between resistance or susceptibility of cattle to development of subclinical PL and serologically defined class I BoLA-A alleles [115]. However, frequencies of BoLA-A alleles diverge considerably between breeds [116–119] and the association was not confirmed at the population level [117]. More sensitive analyses showed that the development of PL was apparently more closely related to class II DRB2 genes [120]. Further characterization of genes within the class II BoLA locus evidenced that the strongest association with resistance or susceptibility to PL corresponded to polymorphisms of the class II DRB3 gene [121,122], the only one actively transcribed among the three DRB genes [123]. Reduced proviral loads were observed in animals carrying the resistance-associated DRB3.2*11 allele, conferring biological significance to this association [124]. These observations were later confirmed and extended to other breeds [125,126]. More recently, a strong linkage between the allele DRB3.2*0902 and genetic resistance to PL was demonstrated in Holstein cows [127]. The presence of DRB3.2*0902 allele correlates with a low proviral load profile (LPL). It was proposed that cattle harboring the *0902 allele and presenting this LPL profile might be unable to transmit BLV in herd conditions [102]. Polymorphisms in the BoLA-DRB3 genes were also correlated with resistance and susceptibility to leukemia/lymphoma in cows [116,126,128,129]. In the experimental sheep model, gene polymorphisms of ovine leukocyte antigen *(OLA)-DRB1* can predict the frequency of tumor development [130].

Besides the polymorphic BoLA-DRB3 gene, other non-MHC genes are also considered to be involved in the development or progression of BLV infection. An association between TNF-alpha and BLV pathogenesis has been established in ovine [131] and bovine species [132]. In the latter, a polymorphism in the promoter region of the TNF-α gene (allele -824G) might contribute to PL susceptibility [132]. Furthermore, polymorphisms in alpha-albumin genes might also be associated with resistance to BLV infection [133].

Selection of BLV resistant animals based on genetic traits faces a series of limitations. First, the relevance and statistical significance of the identified markers must be analyzed at the population level. Large-scale studies in different breeds are required to assess the efficiency and the consequences of selection based on these markers. It is likely that identification of other BoLA polymorphisms or resistance genes will be required to achieve robust and efficient selection. Genetic resistance to BLV infection appears to be a complex mechanism under the control of multiple genes, each contributing slightly to the phenotype [110,134,135]. Poor correspondence between the specific alleles and development of PL or LS [103,122,127] clearly indicates that other viral or host genetic, epigenetic and/or environmental factors contribute to the outcome of infection [136,137]. In this scenario, it might become difficult to prioritize one allele over other/s as an absolute genetic selection marker for selecting BLV resistant cattle [103].

A second limitation is that selection of BLV-resistant animals encounters a major risk due to the narrowing of the genetic pool of the cattle population. This loss of biodiversity is particularly important in resistance to other pathogens [137,138]. On the other hand, selection for BLV resistance may also have adverse effects on beneficial productivity traits. In this sense, it should be mentioned that humoral immune response to other economically important viral diseases (*i.e.*, foot and mouth disease, bovine herpesvirus type 1 and bovine viral diarrhea virus) was not adversely affected in bovines carrying the DRB3.2*0902 allele [139]. Perhaps a more important problem is that selection based on disease resistance might also have adverse effects on productivity traits [134,138].

Due to these limitations, the benefits of selection programs for BLV resistance might not reach a positive benefit:cost ratio.

### 4.4. Epigenetic Modulation of Viral Expression as Therapy

This type of approach issued from fundamental research on viral dynamics in BLV-infected sheep. Conceptually, the host-pathogen interplay during infection is characterized by a very dynamic kinetics resulting in a subtle equilibrium between the virus, which attempts to replicate, and the immune response, which seeks to exert tight control of the pathogen. This equilibrium appeared to be tightly regulated by epigenetic mechanisms. In particular, the extent of chromatin condensation is an essential epigenetic control mechanism determining gene expression and is partly dependent on the level of histone acetylation. In a certainly oversimplified model, chromatin relaxation results from acetylation of the conserved N-terminal histone tails that reduces the interaction with the negatively charged phosphate backbone. This allows unwinding of DNA, leading to gene expression by favoring access of transcriptional activators to their target sites. Conversely, gene silencing or transcriptional repression is the consequence of preventing access of transcriptional activators due to DNA packaging into condensed chromatin. This is mediated by acetyl removal of lysine residues from histone N-terminal tails, restoring a positive charge and increasing the affinity of histones for DNA. The degree of acetylation of the core histones reflects an intrinsic balance between the activities of two families of functionally antagonistic enzymes: histone deacetylases (HDACs) and histone acetyltransferases (HATs), which withdraw or incorporate acetyl groups into core histones, respectively [140–146].

In BLV infected cells, the virus is stably integrated apparently in a transcriptionally silent state [147–154]. Two epigenetic mechanisms, histone acetylation and DNA hypermethylation, correlate with BLV transcriptional repression [149,155–160]. The lack of viral expression in a large proportion of infected cells prevents efficient clearance of the virus by the immune system despite the presence of a vigorous immune response. The rationale of a potential therapy consists in inducing viral expression in provirus-carrying cells, thereby exposing infected cells to the host immune response. A key observation for this working model was that increasing the BLV promoter efficiency paradoxically decreases proviral loads [161]. In this context, a therapeutic approach based on the modulation of host epigenetic mechanisms was proposed to treat BLV infection and disease [159,162,163]. Different HDAC inhibitors (valproate VPA, trichostatin A TSA, trapoxin TPX) efficiently enhance viral transcription directed by the BLV promoter *in vitro* [155,159]. HDAC inhibitors also increase viral expression during *ex vivo* short-term culture of peripheral blood mononuclear cells (PBMCs) from BLV-infected sheep and cattle [155,159]. Consistent with its inhibitory activity, VPA induces hyperacetylation of histone H3 [164]. VPA treatment, in the absence of any other cytotoxic drug, induced tumor regression in BLV-infected sheep and was efficient for leukemia/lymphoma therapy in the sheep model. However, VPA treatment was inefficient in preventing primary infection and reducing PVL in asymptomatic sheep [159].

The efficacy of VPA in BLV-infected cows is presently unknown. However, large scale treatment of BLV infected herds with HDAC inhibitors such as VPA is not justified mainly due to costs. Nevertheless, a treatment with VPA, alone or possibly in combination with antiretroviral drugs (AZT) may still be useful to treat animals with high genetic value.

This strategy might also hold promise for treating adult T cell leukemia (ATL) or human T-lymphotropic virus-associated myelopathy/tropical spastic paraparesis (HAM/TSP), diseases for which no satisfactory treatment currently exists (see Section 5) [165,166]. It is noteworthy that VPA alone had no effect in simian T lymphotropic virus type 1 (STLV-1) infected monkeys but significantly reduced the PVL when combined with AZT [167].

### 4.5. Vaccination

During the last few decades, a series of attempts were performed to develop a vaccine against BLV [11,16,168–170].

#### 4.5.1. Inactivated Virus Vaccines

Early studies evaluated preparations of inactivated BLV obtained from persistently infected cell lines (fetal lamb kidney or FLK, LK15, Bat2Cl1 cell lines) [171–175]. Inactivation was obtained by treatment of the virus with different chemical agents (N-acetylethylenimine, 0.1% Triton X-100, 0.1% formalin, formaldehyde, aminomethylated compounds). These inactivated virus vaccines induced a strong specific neutralizing humoral response and partially protected sheep and cattle from low dose viral challenge [171,172,175]. Protection roughly correlated with the efficiency of the vaccine to induce a strong neutralizing humoral response. However, vaccinated animals became infected with high challenge doses.

#### 4.5.2. Cell-Derived Vaccines

Several tentative vaccines were based on cell lysates from:

- plasma membranes or cell extracts from BLV tumors [176,177].
- BLV-infected FLK or SF-28 cells fixed with 3% glutaraldehyde [178].
- ovine virus non-producing NP2 cells synthesizing only the env gene products (gp51 and gp30) and the capsid protein (p24) of BLV [179,180].

Although some of these trials led to partial protection, this strategy has the intrinsic risk of transmitting infection. Therefore, viral subunits were tested for prophylactic immunization.

#### 4.5.3. Viral Subunit Vaccines

First subunit vaccines were designed with the gp51 surface envelope glycoprotein [178,181–184]. The gp51 sequence is indeed well conserved among BLV isolates [91,185–196] and carries at least three neutralizing epitopes [185,197–203].

A subunit vaccine including purified gp51 obtained from culture supernatant of FLK cells inactivated with 0.01% N-acetylethylenimine was immunogenic but did not consistently protect from BLV challenge [178]. Native viral gp51 glycoprotein obtained from FLK cell culture supernatant absorbed in Al(OH)_3_ also failed to prevent BLV infection in calves [182]. A similar unsuccessful attempt was undertaken with the p24 protein [178].

Based on the ability of anti-BLV antibodies in the colostrum to prevent infection, the three aforementioned conventional approaches were focused on inducing an optimal humoral response [33,39,41,98,99,204–207]. Further experimental evidence supporting the role of the humoral response was obtained by successful immunization of naïve sheep with immunoglobulin G from BLV-infected sheep [208]. It should be emphasized that these early vaccination trials included only few animals and were performed over short periods of time. More importantly, the main concern of these experiments was to determine the correct challenge dose encountered under real herd conditions. On the other hand, a major problem of these vaccines was the fast decline of protective antibody titers. Therefore, the significance of these vaccination strategies in herd conditions remains questionable.

Although important, the humoral response quickly appeared to be insufficient for achieving full protection against BLV infection [209–211]. Additionally, antibody titers showed fast decline in vaccinated animals, an undesired feature in an effective vaccine. Due to these initial failures in vaccination against BLV, more emphasis was given on the cellular component of the immune response.

#### 4.5.4. Recombinant Vaccinia Virus

Recombinant vaccinia virus (RVV) was used as a vehicle for immunization against BLV antigens. RVV is a live recombinant vector having a broad host range specificity, the capacity to carry a large amount of genetic information and, most importantly, the ability to elicit both humoral and cell-mediated immunity.

Preliminary studies with RVV coding for gp51 alone induced neither a humoral response nor protected against BLV in sheep or rabbits [209,212]. In contrast, RVV harboring the complete *env* gene (RVV-*env*) which encodes both gp51 and gp30 glycoproteins produced a neutralizing humoral response in rabbits and sheep [209,210,212–216]. In one study, RVV-*env* failed to induce detectable neutralizing specific antibodies and conferred only partial protection in naïve sheep after challenge with BLV [214]. However, the BLV proviral loads were decreased in vaccinated sheep. An efficient immune response correlated with type 1 helper T cell proliferation and delayed hypersensitivity with predominant involvement of CD8 cytotoxic T lymphocytes [214,215,217]. In two other studies, similar RVV-*env* vaccines induced a strong humoral and CD4+ T cell response and protected against BLV challenge [209,210]. Unfortunately, the RVV-*env* vaccines were inefficient in cows [211,216–218]. Remarkably, vaccination with RVV-*env* in cattle resulted in increasing titers of IgG1 antibodies which might indicate a type 2 response [218,219]. A RVV vector expressing the *gag*, *pol* and *env* genes also failed to protect cattle after challenge [218].

#### 4.5.5. Synthetic Peptides

Short peptides mimicking gp51 B- and T-cell epitopes were tested as potential immunogens [210,220]. Preliminary analyses with peptides encapsulated in mannan-coated liposomes as delivery system induced significant humoral response and specific Th1 type immunity in mice and sheep models [221–223]. A different cocktail that included multiple synthetic peptides of Th, Tc and B epitopes failed to induce protection after challenge despite a cell mediated immune response. In the short term, the peptide vaccine partially reduced BLV replication [184]. Finally, a peptide encompassing a minimal 9-mer CTL epitope of gp51 mixed with Freund’s adjuvant induced a cell-mediated response but did not protect most vaccinated sheep against infection [224,225].

Synthetic peptide-based vaccines showed poor performance possibly due to inadequate stereochemical structure and partial epitope presentation.

#### 4.5.6. DNA Vaccines

DNA vaccines can induce a long-lasting immunity engaging both humoral and cellular components of the immune responses.

A DNA vaccine containing the *env* gene under the control of the cytomegalovirus promoter did not induce a vigorous antibody response but stimulated cellular mediated immunity in vaccinated calves. Partial protection was achieved after BLV challenge [226].

Another DNA vaccine was designed to express the Tax transactivator protein. The Tax vector suppressed BLV replication in immunized sheep likely through a Th1-cell response involving CTL activity [227,228]. However, a more recent study showed that a Tax DNA vaccine elicited a cytotoxic response in the early phase of infection but did not prevent later infection [229]. Neither Tax-specific cytotoxic responses nor the proviral loads were predictive of disease outcome.

As other previously developed immunogens, DNA vaccines were thus disappointing.

### 4.6. Competitive Infection by Attenuated Proviruses

Failure of “traditional vaccines” was likely due to inadequate or short-lived stimulation of all immunity components. Ideally, the optimal vaccine would therefore contain a large number of viral factors permanently stimulating the immune response. Attenuated derivatives of BLV proviruses meet these requirements [169,170,230–237].

Replication-competent BLV proviruses lacking accessory genes and *cis*-acting LTR sequences were designed and evaluated in rats and rabbits [230,231]. A first generation of these genetically simpler viruses was constructed by co-injection of independent vectors encoding *gag*-*pol* and *env* genes. These constructs were devoid of *tax*, *rex*, *R3* and *G4* and contained promoter *cis*-acting regulatory sequences of spleen necrosis virus (SNV). These BLV simpler hybrid derivatives were infectious and induced specific antibodies in a rat model [230,231]. A second type of virus contained *gag*, *pol* and *env* genes in a single genome under the control of SNV regulatory sequences. This viral vector was competent for replication and induced antibody responses against *gag* and *env* structural proteins in rats and rabbits [231,232,235]. While PVL were decreased, the viral vector induced protection against viral challenge in a rabbit model.

Another approach to design attenuated BLV viruses was to delete genes dispensable for infectivity but required for efficient replication [169]. Using an infectious molecular clone [150], a series of BLV mutant or deletant proviruses were engineered and evaluated for infectivity, replication and pathogenicity in sheep. As predicted, large deletions in the structural genes (*gag*, *pol*, and *env*) abrogated infectivity [169,238]. Other specific mutations within the genome (the capsid major homology region, the catalytic sites of the integrase) were deleterious [169,170]. A mutation (nucleotide coordinate 6073) of the immunoreceptor tyrosine-based activation (ITAM) motifs localized in the cytoplasmic tail of the transmembrane gp30 glycoprotein impaired replication but not infectivity. Importantly, the pathogenic potential of the 6073-mutated provirus was significantly reduced but the anti-viral humoral response was preserved [170,233,234,237,239].

Other attenuated BLV proviruses were obtained by deletion of the region expanding from the end of the *env* gene to the splice acceptor site of the *tax/rex* mRNA [169]. These mutants lacking R3 and G4 sequences were infectious but replicated at extremely low levels [240]. Similar conclusions were drawn from HTLV mutant proviruses deleted in the ORFs encoding the p12^I^ and p13^II^/p30^II^ orthologs of R3 and G4 [241–243]. The BLV R3+G4 deletant (pBLVDX) elicited a wild type humoral response but was only very rarely pathogenic in sheep, with a single exception among 20 sheep observed after an unusually long period of latency (7 years) [237].

To summarize, two types of BLV mutants (pBLV6073 and pBLVDX) were infectious, replicated at low levels but elicited a wild-type like immune response. Importantly, these attenuated viruses conferred long-term protection after challenge with a wild-type strain in sheep and partly in cattle [233,234]. Indeed, the idea is that these deleted viruses persistently infect cows and interfere with wild-type BLV propagation in herds. The number of BLV-positive cells is also expected to be reduced in the milk of vaccinated cows, impairing the efficiency of BLV transmission to calves. However, calves born from vaccinated cows with these attenuated mutants would be protected through passive immunization. Whether the attenuated strain is transmitted from cow to calf is presently unknown.

Since attenuated viruses can sometimes be pathogenic, an important issue of these vaccines is biosafety. Pathogenicity can be reduced by combining multiple mutations and deletions that do not affect infectivity but reduce replication. Another risk is conversion of the attenuated vaccine to wild-type. Conceptually, this conversion would only be possible by recombination with another BLV strain. Since the very large majority of the cattle are infected in endemic areas (see Section 3. Epidemiology), the risk is that a fraction of vaccinated animals would become infected with a wild-type virus, a process that occurs anyway with high frequencies. It should also be emphasized that virus recombinations were not observed with attenuated strains even under enforced conditions [233,234].

Trials are currently ongoing to evaluate efficacy and safety of this competitive infection strategy in real herd conditions.

## 5. BLV as a Model for HTLV Therapy and Prevention

BLV and HTLV-1 are closely related deltaretroviruses sharing a similar genomic organization [14,244–246]. Although HTLV and BLV infect different target cells (T and B lymphocytes, respectively) and induce distinct hematological disorders, the two viruses persist, spread and transform through similar mechanisms. A shared feature of their replication strategy is transcriptional silencing, a process that allows escape from the host immune response [147,150,152,153,247]. Both viruses replicate by colonizing new lymphocytes (infectious cycle) as well as by mitosis of the host cell (clonal expansion) [248–250]. A major driving force of viral spread is delivered by the Tax oncoprotein that continuously stimulates proliferation of the infected cell [246,251,252].

In a perspective of comparative virology, the BLV system is a model of pathogenesis for HTLV [170,253–257] (see Table 2). Important outcomes are outlined in this section.

### 5.1. Prevention

In the absence of an effective vaccine, control of HTLV-1 infection relies on prevention practices aiming to reduce viral transmission [258].

#### 5.1.1. Interruption or Short-Term Breast-Feeding

Vertical transmission via postnatal breast-feeding is considered as the most clinically relevant route for HTLV-1 transmission, particularly when practiced over long periods [259–262]. Cell-associated but not free virus in breast milk appears to be the source of infection [263,264]. Consistently, the main preventive measure to decrease the risk of mother to child transmission would be to avoid breast feeding. However, this measure is not always easy to adopt due to behavioral, social and societal reasons. For example, refraining from breast-feeding can lead to social stigma or discrimination and explain why women might have difficulties to follow this recommendation. Furthermore, when sanitary conditions are not met, it might be hazardous to use reconstituted milk with contaminated water. Malnutrition being a primary cause of infant death, poverty can also hamper the use of artificial milk. Finally, there is no optimal substitute for natural milk that provides passive immunity, as described earlier for BLV. Therefore, whilst avoiding or limiting breast-feeding is an effective preventive measure in developed countries where safer alternatives exist, it is frequently not adequate for other regions.

Other strategies also used for BLV include:

- limit the duration of breast feeding (to less than 6 months) [265–267].
- inactivate the virus by heating or freezing-thawing [268–270].

Besides milk, pre- and perinatal infection with HTLV-1 occurs less frequently through transfer of cord blood and placenta syncytiotrofoblastic cells [261,271]. When the PVLs are high, HTLV-1 transmission could be impaired with anti-retroviral drugs such as AZT.

#### 5.1.2. Prevention of Sexual Transmission

Sexual intercourse is another route of HTLV-1 transmission that occurs more efficiently from men to women [272–275]. As for HIV, the use of condoms is the optimal measure preventing HTLV-1 transmission [258].

#### 5.1.3. Prevention of Iatrogenic Transmission

Although less frequent, iatrogenic transmission of HTLV-1 occurs with low efficacy due to needle sharing by intravenous drug users [276–278]. In fact, HTLV-2 appears to be more prevalent in this group [279–282]. Therefore, individuals are strongly recommended to refrain from sharing needles or syringes [258,283]. Iatrogenic transmission of HTLV-1 also occurs in organ transplanted recipients and long-term hemodialyzed patients [284–289].

Parenteral infection by transfusion of contaminated blood is a potential threat for HTLV-1 transmission. Rates of 5–6% infection were found among blood donors of highly endemic areas, such as the Caribbean Islands and Japan [290–292]. In medium prevalence areas of South America, these rates are close to 2% [293–299]. Systematic screening for HTLV-1 in blood banks has become compulsory in many countries. Rapid implementation measures were adopted in highly endemic areas like Japan (1986), Brazil (1993), the Caribbean and the French Isles (1989), as well as in many other developed countries despite low seroprevalence rates (France, USA, Canada, Australia, Netherlands or Denmark). Later on, blood bank screening was applied in developing countries with medium to high seroprevalence among blood donors (Argentina: 2004, Peru: 1999). This policy proved to be successful to decrease HTLV-1 spread by blood transfusion [300–304]. Unfortunately, official screening programs still lack in other regions with high prevalence (Middle East) [305,306], further reflecting differences between industrialized and developing countries.

### 5.2. Vaccination Strategies

Vaccination is probably the best strategy against HTLV-1 infection particularly in endemic areas with high prevalence and in developing economies where other control measures are not implemented [307]. Unfortunately, an efficient, safe and cost-effective vaccine is not available.

As for BLV, an efficient HTLV-1 vaccine should elicit both humoral response and cell-mediated immunity [308]. Focus was also given to the envelope glycoproteins (gp46 and gp21) and the Tax transactivator. Attempts included:

- heat-inactivated HTLV-1 [309];
- *env* glycoproteins produced in *Escherichia coli* [310];
- DNA vaccines encoding *tax* or *env* genes (in combination with RVV-env or RVV-env+gag) [311,312];
- synthetic peptide derived from env gp46 [309,313,314] and Tax [315–319];
- complex chimeric synthetic multivalent peptide vaccines [320–323];
- recombinant vaccinia viruses expressing gag and/or env proteins [312,324–329].

Despite interesting preliminary data, none of these vaccines achieved final development mainly due to partial or short-lived response, safety concerns and perhaps also a lack of interest from an industrial partner. No matter the reasons, a vaccine for HTLV or BLV is currently unavailable and will clearly require significant further development.

#### 5.3. Epigenetic Modulation Strategy and Gene Activation Therapy

In the absence of satisfactory therapy and based on preclinical trials performed in animal models [159,167], gene activation therapy is an option to improve treatment of HTLV-infected patients. As previously described for BLV (see Section 3.4), this approach is designed to activate viral gene expression with VPA in order to expose virus-positive cells to the host immune response [159,256].

In primary cells freshly isolated from HAM/TSP patients, VPA induced hyperacetylation, triggered viral expression and was proapoptotic [159,162,163]. Despite early fluctuations, the PVLs were however not significantly affected after two years of VPA treatment [166]. Importantly, VPA did not alter the anti-viral CTL response and generated only minor side effects. Long term treatment with VPA is thus safe but does not alleviate the condition of HAM/TSP. Further attempts to improve treatment are ongoing. Among these, the combination of VPA and AZT can reduce PVL in STLV-1 infected *P. papio* [167]. If applicable to humans, this treatment could be rapidly translated into clinical therapy and reduce the risk of developing HAM/TSP.

For ATLL, the standard treatment is currently a combination of AZT and alpha-interferon. This regimen is partly efficient for the acute form but unfortunately inoperant for lymphoma ATLL [330,331]. Very recently, an exciting perspective has been presented at the 15th International Conference on Human Retrovirology: VPA combined with AZT and PEG-alpha-interferon induced complete response in 33% of patients associated with molecular remissions (*i.e.*, drop of PVL below threshold levels) [165].

The epigenetic therapy initially developed in the BLV model may thus provide novel opportunities for HTLV-induced diseases.

## 6. Conclusion

In this review, we outlined the currently available approaches to decrease infection rates of BLV: test and eliminate, segregate or manage (see Table 1). Treatment of BLV leukemia with VPA is not economically sustainable except for animals with high genetic value. In the absence of an efficient vaccine, a new strategy based on competitive infection with deletant viruses is presently being evaluated.

Although aims and ethical rules are different for HTLV and BLV, there is an interesting parallelism in prevention and therapeutic measures (see Table 2). An effective vaccine against these viruses is still desperately lacking. Both systems have benefited from each other leading to a better understanding of the mechanisms of viral persistence and pathogenesis.

## Figures and Tables

**Table 1:** Currently available approaches for bovine leukemia virus (BLV) control and prevention.

**APPROACH**	**BASIS OF THE CONTROL PROGRAM**	**ADVANTAGES**	**DISADVANTAGES**
**TEST AND ELIMINATE**	Identify BLV-infected cattle and slaughter positive reactors	Efficient Requires only minimal investment on facilities BLV-free status might be achieved in a relatively short period	May become cost-prohibitive and impracticable depending on the initial prevalence levels Needs constant surveillance Requires official compensatory policies to be successful

**TEST AND SEGREGATE**	Detect and isolate BLV-infected cattle in separate herds Manage separately infected and non-infected cattle in the same housing facilities	Does not need replacement of culled BLV-infected cattle	Needs structural and operational accommodation of infected and non-infected cattle in strictly separated areas Increases costs due to duplicated housing facilities and equipment Requires permanent surveillance Needs long-term commitment to the program

**TEST AND MANAGE**	Take biosafety and management measures to minimize exposure of animals to the infectious agent	Cost-effective Requires only minimal investment on facilities Does not need replacement of culled BLV-infected cattle	Intensively laborious Requires strict adherence to the rigorous implemented measures Needs long-term commitment to the program Susceptible to human and environmental factors Needs adequate training of personnel

**Table 2:** Present and future prevention and treatment strategies for BLV and HTLV-1.

	**BOVINE LEUKEMIA VIRUS (BLV)**	**HUMAN T-CELL LEUKEMIA VIRUS (HTLV-1)**

	**PREVENTIVE MEASURES**	

Avoid or minimize viral transmission through infected-cells present in blood, secretions or excretions	- Use disposable material and individual single-use of needles and syringes - Clean, disinfect or sterilize non-disposable reusable materials, equipment and surgical instruments during dehorning, tattooing, implanting, cauterizing, castration or ear-tagging procedures	- Refrain from sharing needles or syringes with anyone - Avoid donating blood, tissues or organs
Avoid or minimize viral transmission through sexual contact	- Consider natural and/or artificial insemination and embryo transfer with BLV-free donors	- Take precautions to prevent sexual transmission
Avoid or minimize viral transmission through infected-cells present in milk	- Feed calves with colostrum or whole milk from non-infected dams - Use milk replacer or pasteurized colostrum	- Refrain from breast-feeding - Consider short-term breastfeeding (less than 6 months) - Inactivate virus by heating or freeze/thaw procedures

	**VACCINATION**	

	Not available	Not available

	**SELECTION**	

	Not efficient	Not applicable

	**COMPETITIVE INFECTION**	

	Currently tested	Not applicable

	**TREATMENT**	

	VPA but not cost-efficient	AZT+IFN in acute ATL AZT+IFN+VPA in acute ATL and lymphoma? AZT+VPA in HAM/TSP?
